# Nitrous oxide emission by the non-denitrifying, nitrate ammonifier *Bacillus licheniformis*

**DOI:** 10.1186/s12864-016-2382-2

**Published:** 2016-01-19

**Authors:** Yihua Sun, Paul De Vos, Kim Heylen

**Affiliations:** Department of Biochemistry and Microbiology, Laboratory of Microbiology, (LM-UGent), University of Ghent, K.L. Ledeganckstraat 35, 9000 Gent, Belgium; BCCM/LMG Bacteria Collection, K.L. Ledeganckstraat 35, 9000 Gent, Belgium

**Keywords:** Dissimilatory nitrate/nitrite reduction to ammonium (DNRA), Fermentation, Nitrate respiration, Denitrification, Ammonification, Nitrite detoxification

## Abstract

**Background:**

*Firmicutes* have the capacity to remove excess nitrate from the environment via either denitrification, dissimilatory nitrate reduction to ammonium or both. The recent renewed interest in their nitrogen metabolism has revealed many interesting features, the most striking being their wide variety of dissimilatory nitrate reduction pathways. In the present study, nitrous oxide production from *Bacillus licheniformis*, a ubiquitous Gram-positive, spore-forming species with many industrial applications, is investigated.

**Results:**

*B. licheniformis* has long been considered a denitrifier but physiological experiments on three different strains demonstrated that nitrous oxide is not produced from nitrate in stoichiometric amounts, rather ammonium is the most important end-product, produced during fermentation. Significant strain dependency in end-product ratios, attributed to nitrite and ammonium, and medium dependency in nitrous oxide production were also observed. Genome analyses confirmed the lack of a nitrite reductase to nitric oxide, the key enzyme of denitrification. Based on the gene inventory and building on knowledge from other non-denitrifying nitrous oxide emitters, hypothetical pathways for nitrous oxide production, involving NarG, NirB, qNor and Hmp, are proposed. In addition, all publically available genomes of *B. licheniformis* demonstrated similar gene inventories, with specific duplications of the *nar* operon, *narK* and *hmp* genes as well as NarG phylogeny supporting the evolutionary separation of previously described distinct BALI1 and BALI2 lineages.

**Conclusions:**

Using physiological and genomic data we have demonstrated that the common soil bacterium *B. licheniformis* does not denitrify but is capable of fermentative dissimilatory nitrate/nitrite reduction to ammonium (DNRA) with concomitant production of N_2_O. Considering its ubiquitous nature and non-fastidious growth in the lab, *B. licheniformis* is a suitable candidate for further exploration of the actual mechanism of N_2_O production in DNRA bacteria and its relevance *in situ*.

**Electronic supplementary material:**

The online version of this article (doi:10.1186/s12864-016-2382-2) contains supplementary material, which is available to authorized users.

## Background

Denitrification and dissimilatory nitrate/nitrite reduction to ammonium (DNRA) are two key processes, performed by a wide range of *Bacteria* and *Archaea* as well as some *Eukaryotes* [1], responsible for removal of excess nitrate from the environment. Denitrification is the modular step-wise reduction of fixed nitrogen, nitrate or nitrite to a gaseous form, either nitric oxide (NO), nitrous oxide (N_2_O) and/or dinitrogen gas (N_2_). DNRA retains nitrogen in the environment, although N_2_O, contributor to both climate change and ozone depletion in the stratosphere, can also be produced as side product. Comprehensive understanding of the identities and activities of microorganisms as well as cellular mechanisms involved in nitrate removal are crucial for improving models that predict fluxes of nitrate, nitrite and N_2_O [2]. Although several *Firmicutes* have been known for a long time to be nitrate reducers and N_2_O emitters [3–8], their ecological relevance has been minimalized over the past two decades based on molecular community surveys using primers not targeting their divergent denitrification [9–12] or DNRA genes [13] (note that recent primers for DNRA do indeed target *Firmicute* genes [14]). Nevertheless, *Firmicutes* and specifically *Bacillus* can be dominant in ecosystems with important nitrate removal activities such as soil [15], animal manure compost [16] and advanced wastewater treatments [17].

Renewed interest in *Bacillus* has revealed many interesting features like (i) the widespread occurrence of nitrate reduction and denitrification in the genus [18], (ii) the gene inventory for both denitrification and DNRA in one microorganism [19, 20], (iii) a novel type of copper-A-dependent, electrogenic nitric oxide reductase (Cu_A_Nor) [21–24], or (iv) membrane-bound denitrification [25] with a novel organization for the periplasmic nitrate reductase [19, 26]. The most striking observation however is the wide variety of dissimilatory nitrate reduction pathways in members of this genus. The model organism *Bacillus subtilis* uses the cytoplasmic nitrate reductase NarGHI and nitrite reductase NirBD to anaerobically reduce nitrate to ammonium [27, 28], while *Bacillus selenitireducens* produces ammonium via the periplasmic nitrite reductase NrfA [14, 29]. *Bacillus vireti* can do the same but with concomitant N_2_O production via Cu_A_Nor that can be converted to the harmless N_2_ with a NosZ-type reductase [30]. On the other hand, *Bacillus azotoformans* and *Bacillus bataviensis* are canonical denitrifiers, the latter lacking the final reductase, but both also encode the NrfA nitrite reductase, making them potential ammonium producers [19]. In addition, these two organisms demonstrate an unusual high level of gene redundancy, i.e. multiple genes or gene copies encoding the same function (*B. azotoformans* encodes three nitrate, two nitrite, four NO and three N_2_O reductases) [19]. Considering the modularity of denitrification and DNRA, a multitude of enzyme combinations for nitrate reduction are imaginable, even within one microorganism.

*Bacillus licheniformis*, a close relative of *B. subtilis*, is widely distributed as a saprophytic organism in the environment, has numerous commercial and agricultural uses (e.g. production of peptide antibiotics, chemicals and proteases, mitigation of fungal pathogens) and some strains, with abortifacient potential or toxin production, might pose a threat to public health. Certain *B. licheniformis* isolates have been described as denitrifiers [5, 18, 28], mostly based on their ability to produce gas from nitrate anaerobically. Many genomes from *B. licheniformis* have been sequenced and described to date [31–36]. However, their lack of genes encoding either a copper- or cytochrome *cd*_*1*_-dependent nitrite reductase (NirK or NirS respectively), the key enzyme of denitrification, has gone unnoticed, probably because of limited interest in their anaerobic nitrogen metabolism. We have sequenced and analyzed the genomes of three *B. licheniformis* strains previously reported to produce N_2_O [18], and confirmed the lack of *nirS* or *nirK* in their genomes. In addition, physiological data was gathered demonstrating that *B. licheniformis* does not denitrify but is capable of fermentative dissimilatory nitrate/nitrite reduction to ammonium with concomitant production of N_2_O. Both types of data were combined to propose hypothetical pathways for N_2_O production, which present new alternative routes for nitrate reduction and N_2_O production in members of the genus *Bacillus*.

## Methods

### Strains and DNA extraction

*B. licheniformis* LMG 6934, LMG 7559 and LMG 17339 were obtained from the BCCM/LMG bacteria collection. Strains were grown aerobically in trypticase soy broth (TSB) at 37 °C. Cells were harvested after overnight growth and DNA was extracted by the method of Pitcher et al. [37], slightly modified as described previously [38].

### Genome sequencing & annotation

Library preparation and genome sequencing was performed by Baseclear B.V. For sequencing, paired-end strategy on the Illumina Genome Analyzer IIx was used yielding average read lengths of 75 bp for LMG 7759 and LMG 17339 and 50 bp for LMG 6934. Automatic trimming (based on a threshold of Q = 20 and maximum 2 ambiguous bases) and assembly was performed using CLC Genomics Workbench 6.5. The k-mer and bubble size parameters were varied to maximize the N50 and the number of contig of the resulting assembly for each genome. For the consensus sequence, conflicts were resolved by using quality scores and insertion of ambiguity codes. Functional annotation and metabolic reconstruction was performed with the Rapid Annotation Subsystem Technology (RAST) server [39, 40], using RAST gene calling and allowing frame shift correction, backfilling of gaps and automatic fixing of errors. Assigned functions were checked with pBLAST [41] and InterProScan [42]. Missing genes were searched for in the genome with PSI-BLAST using homologous amino acid sequences. The average nucleotide identity (ANI) was calculated with the ANI calculator (http://enve-omics.ce.gatech.edu/ani/) [43].

### Growth experiments

Anaerobic growth experiments were performed in TSB and mineral medium (MM), amended with 10 mM potassium nitrate as electron acceptor. Mineral medium was as described by Stanier et al. [44], including 10 mM phosphate buffer, 2.3 mM (NH_4_)_2_SO_4_, 0.4 mM MgSO_4_•7H_2_O, 0.04 mM CaCl_2_•2H_2_O, 2.3 mM (NH_4_)_2_SO_4_, 27 μM EDTA, 25 μM FeSO_4_•7H_2_O, 10 μM ZnSO_4_•7H_2_O, 25 μM MnSO_4_•H_2_O, 3.8 μM CuSO_4_•5H_2_O, 2 μM Co(NO_3_)2•6H_2_O, 0.196 μM (NH_4_)_6_Mo_7_O_24_•24H_2_O, supplemented with 30 mM glucose as electron donor. Serum vials (120 ml) were rinsed with 1 M HCl overnight to remove growth inhibiting substances, and subsequently washed four times and rinsed with distilled water before use. Serum vials with 50 ml medium were sealed with black butyl-rubber stoppers. After autoclaving, the headspace of the serum vials was replaced via five cycles of evacuating and refilling with helium. Serum vials were inoculated (1 % v/v) with a suspension of optical density OD_600_ of 1.0 ± 0.05. Each growth experiment was performed in triplicate and non-inoculated media in duplicate were included to check for potential nitrosation reactions in sterile medium. After inoculation, serum vials were incubated at 37 °C, 150 rpm, for 72 h. Preliminary end-point analyses demonstrated that all three strains did not produce N_2_ (later confirmed by absence of *nosZ* gene from the genomes, see further) so their dissimilatory nitrate reduction metabolism was explored without addition of acetylene to the headspace. Statistical differences in growth rate and yield of LMG 6934 between TSB and TSB amended with 10 mM nitrate were assessed using the independent t-test after Levene’s test for equality of variances, and main and interaction effect of medium and strain on end-product concentration using factorial ANOVA and Least Significant Difference post-hoc testing in SPSS23.

### Analytical procedures

Samples of 1 ml were taken from cultures through the rubber septum of serum vials with sterile syringes for growth determination and colorimetric determination of ammonium, nitrate and nitrite. Growth was determined by measuring the optical density OD_600_ of 100 μl sample in duplicate in microtiter plates and standardized to 1 cm pathlength using PathCheck Sensor of the spectrophotometer (Molecular Devices, Spectramax plus 384, USA). For colorimetrics, 500 μl from remaining sample was pretreated with 2.5 ml of 2 M potassium chloride by shaking 1 h at 150 rpm and subsequent filtration (0.2 μm) to extract inorganic nitrogen and remove interfering compounds. Filtered samples were centrifuged at 13000 rpm for 2 min to remove the cells and kept frozen at -20 °C until colorimetric determination. Ammonium concentration was determined with the salicylate-nitroprussidine method (absorption at a wavelength of 650 nm) [45], nitrite and nitrate concentrations were determined with Griess reaction [46] and Griess reaction with cadmium [47, 48] respectively. For end-point measurements, ammonium production was corrected per strain for the amount of ammonium assimilated based on OD_600_ values obtained. Standard curves covered ranges suitable for the tested media and were strictly linear with an R_2_ of 0.99. For determination of N_2_O, 1 ml sample of the headspace of serum vials was taken with sterile syringes, and was injected into the gas chromatograph (Compact GC with EZChrom Elite Software, Interscience, Netherlands, 2012). N_2_O concentrations were corrected for pressure and solubility based on Henry’s law.

### Accession numbers

The Whole Genome Shotgun projects of *B. licheniformis* LMG 6934, LMG 7559 and LMG 17339 have been deposited at DDBJ/EMBL/GenBank under the accession numbers AZSY00000000, AZSX00000000, and AZSZ00000000 respectively. The versions described in this paper are the first versions.

## Results

### Dissimilatory nitrate reduction metabolism

Three genotypically distinct *B. licheniformis* strains (Coorevits, A. & De Vos, P., personal communication) from various origins were selected for determination of their dissimilatory nitrate reduction metabolism based on a previous study that demonstrated their capacity to produce N_2_O [18]. *B. licheniformis* LMG 6934 was originally isolated from garden soil, LMG 7559 from flour and LMG 17339 from silage.

**Fig. 1:**
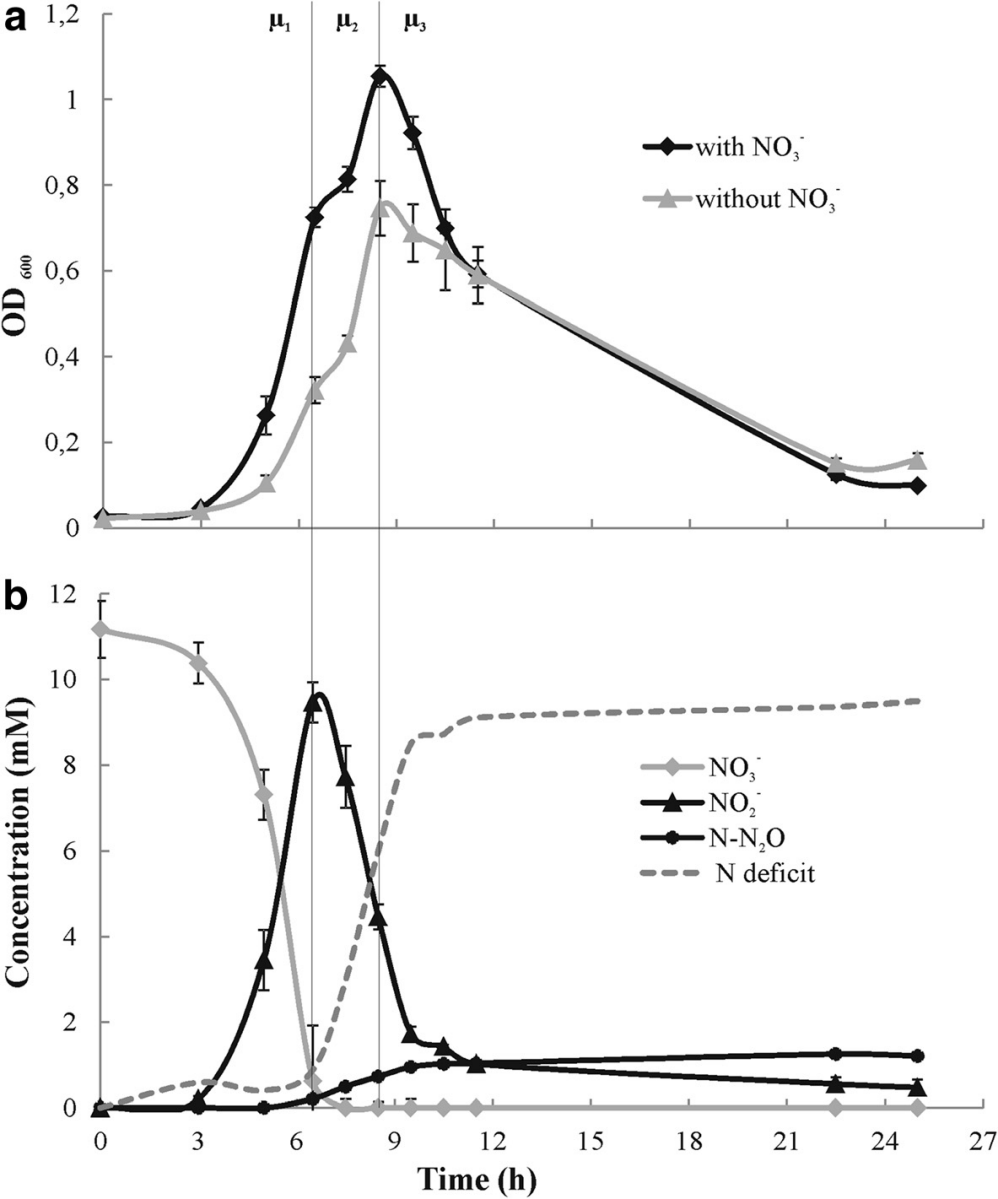
Anaerobic growth (OD_600_) *of B. licheniformis* LMG 6934 in TSB (a) and nitrate, nitrite and N_2_O concentrations (mM)(b) over time. Error bars show standard deviation (*n* = 3). Different growth phases based on primary metabolism in TSB amended with nitrate are marked: μ_1_, respiratory growth with nitrate as electron acceptor; μ_2_, fermentative growth after nitrate is depleted; μ_3_, sporulation. Dashed curve visualizes N deficit caused by the lack of ammonium data

Growth of LMG 6934 (Fig. 1a) under anaerobic headspace in TSB amended without and with nitrate (11 mM) commenced after a short lag phase of approximately 3 h, a steep exponential phase followed, with maximal growth achieved after 8.5 h, after which cells sporulated very quickly without a stationary phase (Fig. 1a). Between 6.5 and 7.5 h, growth slowed down probably due to a depletion of the preferential carbon source in the medium with a shift to another electron donor, as this was observed both for fermentative and respiratory growth. The anaerobic growth rates were comparable with and without nitrate as electron acceptor (μ_nitrate_/h = 0.189 ± 0.004 h^−1^ and μ_ferm_ = 0.179 ± 0.011 h^−1^ (*p* = 0.264)), but with significantly different maximal growth yield (OD_600_ of 1.05 ± 0.02 with nitrate and 0.75 ± 0.06 without nitrate (*p* = 0.002)) reflecting the different ATP yield of a respiratory and a fermentative life style. In the presence of nitrate (Fig. 1b), growth was initially supported by nitrate reduction (μ_1_ = 0.19 ± 0.004 h^−1^), with concomitant nitrite production. All nitrate was converted to nitrite but the maximal nitrite peak was probably missed between 5 and 6.5 h of incubation, which was deduced from the onset of N_2_O production (0.2 mM N-N_2_O at 6.5 h). When nitrate was almost depleted (at 6.5 h, 0.62 mM residual nitrate), nitrite was reduced (μ_2_ = 0.16 ± 0.02 h^−1^), which continued after maximal growth at 8.5 h was achieved and sporulation had started (μ_3_). This suggested that nitrite reduction did not support growth during the μ_2_ phase, but rather fermentation was responsible for growth after nitrate depletion. During nitrite reduction, a continuous increase in N_2_O was observed, with a maximum of 1.3 ± 0.07 mM N-N_2_O at the end of the incubation (accounting for 12 % of all reduced nitrite), and 0.5 ± 0.18 mM of nitrite remaining in the medium (Fig. 1b). Due to technical constraints and interference of amines from degradation of proteins in the TSB during bacterial growth, ammonium was not monitored during these growth experiments.

**Fig. 2:**
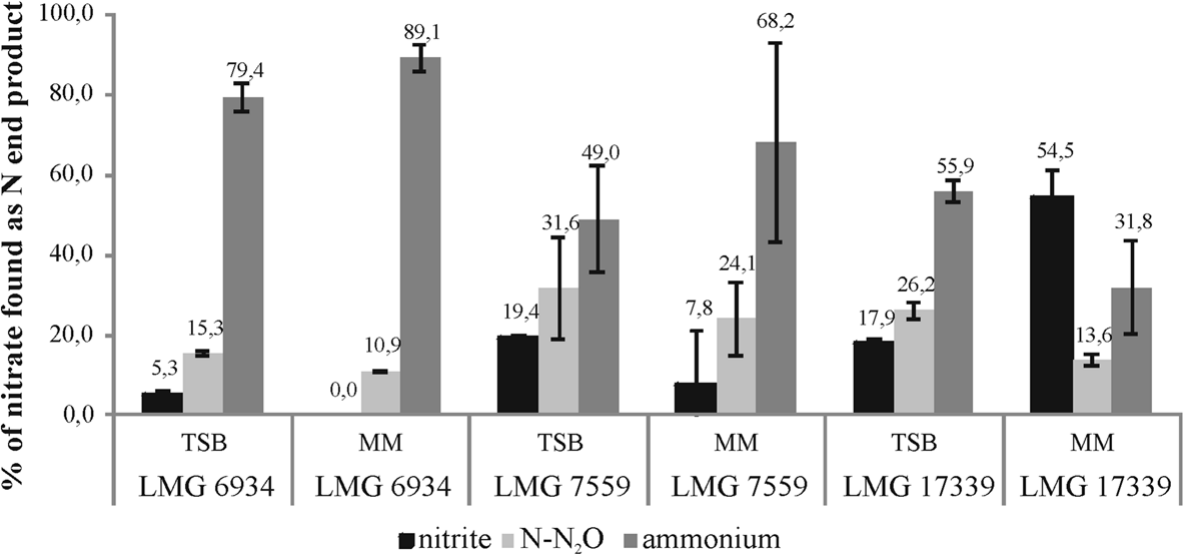
Percentages of end-products of anaerobic nitrate reduction by *B. licheniformis* strains in TSB and mineral medium with 30 mM glucose (MM), amended with 10 mM nitrate. Error bars represent standard deviation (*n* = 3). The larger error bars for LMG 7559 result from differences in one of the three replicates. Measured concentrations of ammonium were corrected for loss through assimilation. N deficit in TSB experiments was attributed to ammonium production, which could not be measured, and visualized as such for convenience of comparison

To compare the dissimilatory nitrate reduction metabolism of LMG 6934 with those of LMG 7559 and LMG 17339 and to confirm ammonium production from nitrite, end-point experiments after a 72 h-incubation in anaerobic conditions were performed in TSB and mineral medium with 30 mM glucose, both amended with nitrate. Maximal growth of LMG 7559 and LMG 17339 was achieved within 11 h, again immediately followed by a rapid sporulation (data not shown). In mineral media with glucose, most nitrate was converted to ammonium (31.8 to 89.1 %; Fig. 2), confirming the ammonium-producing capacity of all three strains (for TSB, the nitrogen deficit was attributed to ammonium production, which could not be measured). A significant strain effect on the ratios of end-products was observed (*p* ≤ 0.008) (Fig. 2), which after decomposition appeared to be mostly attributed to differences in ammonium and nitrite concentrations. In addition, the amount of N-N_2_O produced from nitrate was substantially lower in mineral medium than in TSB for all strains (15.8–32.9 % for TSB vs 10.9–24.1 % in MM) (*p* = 0.023). Only for LMG 17339, the medium also had a significant effect on ammonium and nitrite (*p* ≤ 0.024). Both strain and medium effect were quite unexpected, as the three strains are closely related and the major carbon source in TSB, namely glucose, is also used in the mineral medium.

### Genome analyses

Draft genomes of the three *B. licheniformis* strains were obtained, the genome statistics are given in Table 1. The gene inventory for assimilatory and dissimilatory nitrate reduction and related transport and regulation was almost identical for the three genomes (Table 2). Details will be given for LMG 6934 and differences with LMG 7559 and LMG 17339 will be highlighted.

**Table 1:** Genome characteristics of three analyzed *B. licheniformis* genomes. Number of coding sequences is based on annotation obtained via RAST

	LMG 6934	LMG 7559	LMG 17339
# contigs (# bp)	53 (4,138,686 bp)	69 (4,341,862 bp)	80 (4,333,151 bp)
N_50_	654,545	123,311	102,733
av. read coverage	87.4	82.3	233.7
% G + C	45.9	45.8	46.1
RNA	1 rRNA operon 30 tRNA	1 rRNA operon 30 tRNA	1 rRNA operon 36 tRNA
# coding sequences	4576	4559	4425
accession number	AZSY00000000	AZSX00000000	AZSZ00000000

**Table 2:** Overview of gene inventory involved in nitrogen metabolism, transport and regulation of *Bacillus licheniformis*

function	protein	gene	locus_tag (gene coordinates)		
			LMG 6934	LMG 7559	LMG 17339
dissimilatory nitrate reduction to nitrite	respiratory nitrate reductase subunit alpha	*narG1*	LI6934_10240 (contig17_44191_40505)	LI7559_10220 (contig17_109260_112943)	LI17339_03250 (contig01_602796_599110)
		*narG2*	LI6934_11815 (contig19_3766_7449)	LI7559_18375 (contig29_333921_330235)	-
	respiratory nitrate reductase subunit beta	*narH1*	LI6934_10235 (contig17_40515_39046)	LI7559_10225 (contig17_112933_114483)	LI17339_03245 (contig01_599120_597651)
		*narH2*	LI6934_11820 (contig19_7439_8989)	LI7559_18370 (contig29_330245_328776)	-
	respiratory nitrate reductase subunit delta	*narJ1*	LI6934_10230 (contig17_39028_38486)	LI7559_10230 (contig17_114470_115018)	LI17339_03240 (contig01_597632_597090)
		*narJ2*	LI6934_11825 (contig19_8976_9524)	LI7559_18365 (contig29_328758_328216)	-
	respiratory nitrate reductase subunit gamma	*narI1*	LI6934_10225 (contig17_38489_37803)	LI7559_10235 (contig17_115039_115740)	LI17339_03235 (contig01_597093_596407)
		*narI2*	LI6934_11830 (contig19_9545_10246)	LI7559_18360 (contig29_328219_327533)	-
assimilatory nitrate/nitrite	assimilatory nitrate reductase large subunit	*nasC*	LI6934_20135 (contig48_34712_36769)	LI7559_01055 (contig02_88956_91013)	LI17339_17560 (contig10_88671_90728)
reduction to ammonium	assimilatory nitrite reductase [NAD(P)H] large subunit	*nirB*	LI6934_20140 (contig48_36883_39303)	LI7559_01060 (contig02_91127_93547)	LI17339_17565 (contig10_90842_93262)
	assimilatory nitrite reductase [NAD(P)H] small subunit	*nirD*	LI6934_20145 (contig48_39334_39654)	LI7559_01065 (contig02_93578_93898)	LI17339_17560 (contig10_93293_93613)
transporters	ammonium transport	*amt1*	LI6934_11075 (contig18_87147_88526)	LI7559_21025 (contig40_60895_59966)	LI17339_05915 (contig03_429269_428058)
		*amt2*	LI6934_06945 (contig13_69958_71169)	LI7559_12245 (contig20_86027_87406)	LI17339_15165 (contig06_266544_265900)
	nitrate/nitrite transporter (NarK2-type)	*narK1*	LI6934_10275 (contig17_50582_49398)	LI7559_10215 (contig17_107711_109207)	LI17339_03285 (contig01_609186_608002)
		*narK2*	LI6934_11810 (contig19_2217_3713)	LI7559_18410 (contig29_340309_339125)	LI17339_16960 (contig09_16054_161849)
		*narK3*	LI6934_04585 (contig09_42906_44111)	LI7559_1255 (contig21_43506_44711)	-
	formate/nitrite transporter	*nirC*	LI6934_08170 (contig13_306187_306975)	LI7559_03215 (contig06_176423_177211)	LI17339_04610 (contig03_178114_177326)
NO reduction to N_2_O	NO reductase large subunit-like protein	*qnorB*	LI6934_10290 (contig17_51910_54264)	LI7559_18425 (contig29_341636_343990)	LI17339_03300 (contig01_610516_612870)
	NO reductase activation protein	*norD1*	LI6934_02700 (contig04_119995_118082)	LI7559_00105 (contig01_24308_22395)	LI17339_13375 (contig04_735870_736760)
		*norD2*	LI6934_02705 (contig04_120896_120006)	LI7559_00110 (contig01_25209_24319)	LI17339_13380 (contig04_736770_738683)
detoxification	flavohemoglobin	*hmp1*	pLI6934_11805 (contig19_1907_689)	LI7559_10210 (contig17_107401_106181)	LI17339_14225 (contig06_89281_88067)
		*hmp2*	LI6934_03825 (contig07_12629_11415)	LI7559_14100 (contig23_84124_85338)	-
	NO synthase	*nos*	LI6934_16440 (contig34_14900_15997)	LI7559_14725 (contig27_17226_16129)	LI17339_20905 (contig20_78781_79878)
regulation	P_II_-type signal transduction protein	*glnK1*	LI6934_11070 (contig18_86749_87090)	LI7559_12240 (contig20_85629_85970)	LI17339_05910 (contig03_428039_427689)
		*glnK2*	LI6934_06950 (contig13_71188_71538)	LI7559_21020 (contig40_59850_59500)	LI17339_15170 (contig06_267014_266677)
	global nitrogen regulator	*tnrA*	LI6934_15560 (contig31_18775_18443)	LI7559_22170 (contig47_44732_45064)	LI17339_00475 (contig01_88311_87979)
	NO-dependent regulator DnrN or NorA	*dnrN*	LI6934_10295 (contig17_55022_54309)	LI7559_18430 (contig29_344748_344035)	LI17339_03305 (contig01_613628_612915)
	Nitrite-sensitive transcriptional repressor of NO stress response	*nsrR*	LI6934_03830 (contig07_12887_13318)	LI7559_14095 (contig23_83866_83432)	LI17339_14220 (contig06_87809_87378)
	Nitrate/nitrite sensor protein	*narX1*	LI6934_03855 (contig07_16463_15315)	LI7559_14090 (contig23_83082_83402)	LI17339_14195 (contig06_84234_85382)
	Nitrate/nitrite response regulator protein	*narL1*	LI6934_03850 (contig07_15327_14677)	LI7559_02755 (contig06_92319_91687)	LI17339_14200 (contig06_85379_86023)
		*narL2*	LI6934_07715 (contig_13_222155_221523)	LI7559_14070 (contig23_80295_81443)	-
	transcriptional regulator Crp/Fnr	*fnr1*	LI6934_10270 (contig17_49251_48538)	LI7559_18405 (contig29_338978_338265)	LI17339_03280 (contig01_607856_607143)
		*fnr2*	LI6934_10285 (contig17_51086_51754)	LI7559_18420 (contig29_340813_341487)	LI17339_03295 (contig01_609691_610359)

**Fig. 3:**
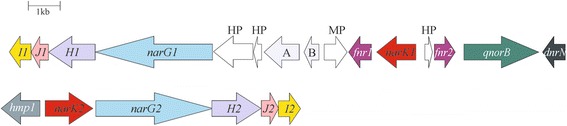
Physical map of *B. licheniformis* LMG 6934 and LMG 7559 *nar* gene clusters and their genome environment. *Arrows* show the direction of transcription. Open reading frames are drawn to scale. Homologous genes are shown in identical colors. Note that LMG 17339 only contains nar1 gene cluster with identical genome environment except for an extra HP immediately upstream of *narG1*. HP, hypothetical protein; MP, membrane protein; A, gene for the radical SAM domain heme biosynthesis protein; B, gene for a probable transcription regulator arfM

The genome of LMG 6934 contained two copies of the *nar* operon (*narGHJI*) coding for the cytoplasmic, membrane-bound nitrate reductase (Table 2; Additional file 1: Figure S1). The two NarG sequences are quite divergent, only sharing 53.7 % amino acid sequence identity. The *nar1* operon (Fig. 3) is located in a gene cluster with downstream the genes for the anaerobic regulatory protein Fnr (Fumarate-Nitrate reductase Regulation) (*fnr1*), a NarK2-type low-affinity nitrate/nitrite antiporter (*narK1*) (Additional file 1: Figure S2), a second Fnr (*fnr2*), a quinol-dependent NO reductase (*qnorB*) and a NO-dependent regulator (*dnrN*). The *nar2* operon (Fig. 3) is immediately downstream of CDS for a second NarK2-type nitrate/nitrite antiporter (*narK2*) (Additional file 1: Figure S2) and a flavohemoprotein (*hmp1*). The two-component nitrate/nitrite sensor regulator system (*narXL*) is encoded downstream of the genes for a nitrite-sensitive transcriptional repressor of NO stress response (*nsrR*) and a second flavohemoprotein (*hmp2*), while genes for a third NarK2-type nitrate/nitrite antiporter (*narK3*), a formate/nitrite transporter (*nirC*), a second *narL* copy NO reductase activation proteins (*norDQ*), and the global nitrogen regulator (*tnrA*) are found separate on the genome. The gene for NO synthase (*nos*) was also found. Two genes encoding a putative NorV, a flavorubredoxin that could be capable of detoxification of NO to N_2_O [49, 50], were also found. However, no gene for the associated oxidoreductase NorW or regulator NorR were found down- and upstream respectively, suggesting that NorV is unlikely to be functional as NO reductase. Nevertheless, all features for nitrate sensing, transport, reduction to nitrite and its regulation are found, as well as for NO reduction to N_2_O. In addition, related to nitrogen assimilation, the operon for assimilatory nitrate and nitrite reduction and two genes for AmtB-type ammonium transporters with each upstream the regulatory gene *glnK* are found. Notably, genes for a NirS- or NirK-type nitrite reductase to NO, a NosZ-type N_2_O reductase, or a Nrf-type nitrite reductase to ammonium are absent from the genome. The gene inventory and organization for LMG 7559 was identical to LMG 6934 (Table 2). Note that strain LMG 7559 is equivalent to ATTC 9945, for which a complete genome sequence has already been published since the start of our genome analyses [33]. For clarity, both genomes will be included in the remainder of the genome analyses. The genome of LMG 17339 only contained one *nar* operon, two NarK2-type nitrate/nitrite antiporters and one copy of *hmp* and *narL* (associated with *narX*), but for the remainder was identical in gene content and organization to LMG 6934 (Table 2).

Whole genome clustering based on the peptidome content [51, 52], in which the amino acid sequences of a genome are converted to tryptic peptides, i.e. the tryptic peptidome, of all publically available *B. licheniformis* genomes (dd June 2014) confirmed the two generally accepted distinct lineages within *B. licheniformis*, BALI1 and BALI2 [53] (Additional file 1: Figure S3). Average nucleotide identities (ANI) of the genomes from the strains within BALI1 (99.70 % ± 0.03) and BALI2 (98.92 % ± 0.03) were well-above the arbitrary 94–95 % cut-off criterion for species delineation, while between group ANI values were ambiguous (94.24 % ± 0.07) [54, 55]. Interestingly, comparison of the NarG sequences of the three *B. licheniformis* genomes from this study, all other publically available *B. licheniformis* genomes and representatives of other *Bacillus* species showed two distinct clusters, each supported by high bootstrap values (Additional file 1: Figure S1). NarG1 from LMG 6934, NarG2 from LMG 7559 and NarG from LMG 17339 grouped within the BALI 1 cluster, consisting of sequences derived only from *B. licheniformis* and one *Bacillus* sp. NarG2 from LMG 6934 and NarG1 from LMG 7559 fell within cluster BALI2, which also included *B. bataviensis* and one of the two NarGs from *B. azotoformans*. In addition, all *B. licheniformis* genomes from BALI1 consistently harbored only one copy of the *nar* operon, two copies of *narK* and one copy of *hmp*, in contrast to those from BALI2 with two *nar* operons, three *narK* copies and two *hmp* copies (data not shown).

## Discussion

### General metabolism

Strains belonging to the species *B. licheniformis* have often been considered as denitrifiers [5, 18], based on their ability to produce gaseous end-products specifically N_2_O from nitrate. However, our analyses demonstrated that *B. licheniformis*, like *B. subtilis* [27, 28], is capable of nitrate respiration and fermentative dissimilatory nitrite reduction to ammonium rather than denitrification. All currently available *B. licheniformis* genomes lack a *nirK*- or *nirS*-type nitrite reductase and growth experiments with three strains confirmed that nitrogen gasses were not produced in stoichiometric amounts. *B. licheniformis* first reduced nitrate to nitrite (Fig. 1, μ1), using the cytoplasmic NarGHI, to support growth accumulating high levels of nitrite before subsequently switching to fermentation after nitrate got depleted. During fermentation (Fig. 1, μ2), ammonium was produced from nitrite, probably using the NADP-dependent nitrite reductase NirBD (also called NasDE) that can serve for both assimilation and dissimilation. For *B. subtilis* it is still undetermined if nitrite reduction is coupled to energy production through proton motive force [28]. However, since nitrite reduction and production of N_2_O continued after growth had ceased, as described before for other non-denitrifying N_2_O producers [30, 56, 57], these N conversion seem unrelated to energy conservation. Nevertheless, during fermentative growth, DNRA can serve as an electron-sink allowing re-oxidation of NADH with the generation of one extra ATP by substrate level phosphorylation for each acetate produced [58, 59].

### Hypothesis for NO and N_2_O formation

**Fig. 4:**
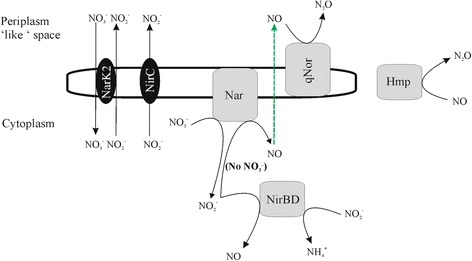
Proposed hypothetical pathways for anaerobic nitrogen reduction in *B. licheniformis*. Schematic representation of enzymes are given in grey, transporters in black. Diffusion of gaseous NO through cytoplasmic membrane is indicated by the dashed arrow. Soluble Hmp can be located in the cytoplasm and periplasm

To our knowledge, NO or N_2_O production and associated cellular mechanisms have never been described for the model organism *B. subtilis*. Therefore, we built on the knowledge from other model organisms to deduce plausible hypotheses to explain our observations (Fig. 4). Nitrite conversion to NO in *E. coli* was shown to occur only after nitrate was depleted, in presence of molybdate - the cofactor of NarGHI -, continued in *nirB*-mutants [60] but was absent in *narG*-mutants [61], suggesting that the NarGHI had a double function and converted nitrite to NO. Also in *Salmonella enterica* serovar *Typhimurium*, NarGHI was unequivocally responsible for NO generation from nitrite, which was completely eliminated in a *narGHI* mutant [62]. Later mutagenesis experiments in *E. coli* could not confirm the involvement of the cytoplasmic nitrate reductase in NO evolution, probably because the experiments were conducted in the absence of nitrate and thus lacked nitrite formed from NarGHI activity during growth [63]. Rather NirB and NrfA, besides their primary role converting nitrite to ammonium, appeared to be involved in NO production [63], with their relative importance dependent on the nitrite concentration [64, 65]. But, in contrast to *B. vireti* capable of DNRA in combination with NosZ-mediated N_2_O reduction [30] but similar to *B. subtilis*, *B. licheniformis* lacks a *nrfA* gene. Furthermore, the activity of NO synthase (NOS), which produces NO from arginine as a defense mechanism against oxidative stress under aerobic conditions in *B. subtilis* [66], is unlikely under our hypoxic test conditions, making the involvement of NarGHI or NirB in the generation of NO from nitrite most plausible (Fig. 4).

Next, NO homeostasis is crucial to limit the toxicity of NO, which is a reactive nitrogen species capable of damaging bacterial proteins, lipids and DNA, and binding to metal centers. NO can freely diffuse through the membrane and can be converted to N_2_O in the periplasmic-like space by the quinol-dependent NO reductase qNor (Fig. 4). This reductase is known to be present both in denitrifiers and non-denitrifiers [67–70], including pathogenic bacteria where it is part of their defense mechanism against nitrosative stress. In addition, *B. licheniformis* genomes also encode the flavohemoglobin Hmp. Hmp, found both in cytoplasm and periplasm [71], is known to convert NO to nitrate aerobically and to N_2_O anaerobically [72, 73]. However, as the latter conversion is at greatly reduced activity [74], it is unsure whether this enzyme is relevant for NO detoxification in *B. licheniformis* with qNorB; indeed *hmp* appeared not to upregulated in anaerobic conditions at high levels of nitrite in *B. vireti* that contained a copper-dependent NO reductase type 1 [30]. The periplasmic NrfA [75, 76] and the cytoplasmic flavorubredoxin NorV and its associated oxidoreductase NorW [49, 50] that can both anaerobically reduce NO to ammonium and/or N_2_O, are not found in *B. licheniformis*. Notably, the gene inventory for anaerobic nitrate and nitrite metabolism in *B. licheniformis* and *B. subtilis* only seems to differ in the presence of a *qnorB* gene in the former organism, making it likely that *B. subtilis* is capable of NO production. This was hinted at by micromolar range N_2_O production by *B. subtilis* 1A01 [77] for which the genome is unfortunately not available. Mutagenic and transcriptomic studies are necessary to confirm our hypothetic pathways for NO and N_2_O production in *B. licheniformis*.

### N end-products: environmental significance and microdiversity

Non-denitrifying nitrate reducers, mostly belonging to *Enterobacteriaceae* or *Bacillaceae*, have been reported to reduce typically about 5 to 10 % of nitrate to N_2_O, with sometimes high quantities up to 35 %, which evolved mostly after growth has ceased [30, 56, 57, 65, 77]. For *B. licheniformis,* measured N_2_O production from nitrate was within these ranges but was nevertheless quite substantial, with up to one-third of all nitrate converted to N_2_O (Fig. 2). How environmentally relevant these N_2_O emissions from non-denitrifiers are remains difficult to establish without ways to differentiate them from denitrification; in isotope pairing experiments, non-denitrifiers will also produce ^30^N-N_2_O. In addition, even with mechanistic understanding, deducing specific target genes for molecular surveys will be nearly impossible, as different mechanisms have already been described for a single microorganisms like *E. coli* [78], *S. thyphimurium* [62] and *B. vireti* [30] and the genes involved have dual functions, e.g. NarG, NirB and NrfA.

Despite high N_2_O emission, most nitrite was indeed converted to ammonium (12.2 – 51.0 %), which is in agreement with previous observations for pure cultures under nitrate limitation (valid for both growth conditions applied here as growth continued via fermentation after nitrate depletion) [30, 56, 77]. Interestingly, the ratio of end-products from nitrate varied quite substantially between all three *B. licheniformis* strains. However, as differences were also apparent between LMG 6934 and LMG 7559, this phenotypic heterogeneity could not be linked the specific gene duplications in BALI2 genomes. An alternative explanation might be distinct regulatory motifs in the promotor regions of the genes involved, although the same regulatory genes were encoded in the three genomes with identical relative genome locations (Table 2). It is long been accepted that closely related bacteria do not necessarily share the capacity to denitrify, and even when they do, can have different denitrifying phenotypes. However, our data suggest that phenotypic heterogeneity or niche differentiation between closely related strains, which has recently been reported for N_2_O production in *Bacillus* [79], *Thauera* [80] and *Methylomonas* [81], might not always be linked to genetic variation.

## Conclusions

Using physiological and genomic data we have demonstrated that the common soil bacterium *B. licheniformis* does not denitrify but is capable of fermentative dissimilatory nitrate/nitrite reduction to ammonium with concomitant production of N_2_O. Based on the genomic inventory, alternative routes for N_2_O production, similar to those in *Enterobacteriaceae* and thus far unreported in bacilli, were proposed. Significant strain-dependent differences were found between three closely related strains that could not be linked to genetic features. Considering its ubiquitous nature and non-fastidious growth in the lab, *B. licheniformis* is a suitable candidate for further exploration of the uncertainty of the mechanism of N_2_O production in DNRA bacteria and its relevance *in situ*.

### Availability of supporting data

All the supporting data are included as additional files.

## Additional file

Additional file 1: Figure S1. Phylogenetic tree of full-length NarG. Sequences were taken from genomes included in the manuscript as well as all from publically available B. licheniformis genomes (dd June 2014) and genomes from other Bacillus species, protein ID or locus tag is given between brackets. The evolutionary history was inferred using the Neighbor-Joining method. The optimal tree with the sum of branch length = 1,68972248 is shown. The percentage of replicate trees in which the associated taxa clustered together in the bootstrap test (500 replicates) are shown next to the branches. The evolutionary distances were computed using the Poisson correction method and are in the units of the number of amino acid substitutions per site. All positions containing gaps and missing data were eliminated. There were a total of 1094 positions in the final dataset. Evolutionary analyses were conducted in MEGA6. **Figure S2.** Phylogenetic tree of full-length NarK. Sequences were taken from several Bacillus species, as well as reference genomes, locus tag is given between brackets. The evolutionary history was inferred using the Neighbor-Joining method. The optimal tree with the sum of branch length = 6,03046656 is shown. The percentage of replicate trees in which the associated taxa clustered together in the bootstrap test (500 replicates) are shown next to the branches. The tree is drawn to scale, with branch lengths in the same units as those of the evolutionary distances used to infer the phylogenetic tree. The evolutionary distances were computed using the Poisson correction method and are in the units of the number of amino acid substitutions per site. All positions containing gaps and missing data were eliminated. There were a total of 358 positions in the final dataset. Evolutionary analyses were conducted in MEGA6. **Figure S3.** Whole genome clustering based on a similarity matrix using the peptidome content, in which amino acid sequences of a genome are converted to tryptic peptides, i.e. the tryptic peptidome. All publically available B. licheniformis genomes (dd June 2014) were included and are designated by their strain number: 9945A (accession number NC_021362), ATCC 14580 (accession number NC_006270), DSM 13 (=ATCC 14580; accession number NC_006322), 5-2-D (accession number NZ_AJLW01000000), F1-1 (accession number NZ_AZSL01000000), 3F-3 (accession number NZ_JFYM01000000), F2-1 (accession number NZ_AZSM01000000), WX-02 (accession number NZ_JH636050), 10-1-A (accession number NZ_AJLV01000001), CGMCC 3963 (accession number NZ_AMWQ01000000), CG-B52 (accession number NZ_AVEZ01000000). Analyses were performed using the Peptidome tool (http://unipept.ugent.be/). (DOCX 29 kb)

## Abbreviations

DNRA: Dissimilatory nitrate/nitrite reduction to ammonium
TSB: Trypticase soy broth
RAST: Rapid annotation subsystem technology
ANI: Average nucleotide identity
MM: Mineral medium
OD: Optical density
