# Supplementary material for: Nitrous oxide emission by the non-denitrifying, nitrate ammonifier Bacillus licheniformis
Source: BMC Genomics. 2016 Jan 19;17:68. doi: 10.1186/s12864-016-2382-2 (PMC4719734; doi:10.1186/s12864-016-2382-2)
Supplement: Additional file 1: Figure S1. — Phylogenetic tree of full-length NarG. Sequences were taken from genomes included in the manuscript as well as all from publically available B. licheniformis genomes (dd June 2014) and genomes from other Bacillus species, protein ID or locus tag is given between brackets. The evolutionary history was inferred using the Neighbor-Joining method. The optimal tree with the sum of branch length = 1,68972248 is shown. The percentage of replicate trees in which the associated taxa clustered together in the bootstrap test (500 replicates) are shown next to the branches. The evolutionary distances were computed using the Poisson correction method and are in the units of the number of amino acid substitutions per site. All positions containing gaps and missing data were eliminated. There were a total of 1094 positions in the final dataset. Evolutionary analyses were conducted in MEGA6. Figure S2. Phylogenetic tree of full-length NarK. Sequences were taken from several Bacillus species, as well as reference genomes, locus tag is given between brackets. The evolutionary history was inferred using the Neighbor-Joining method. The optimal tree with the sum of branch length = 6,03046656 is shown. The percentage of replicate trees in which the associated taxa clustered together in the bootstrap test (500 replicates) are shown next to the branches. The tree is drawn to scale, with branch lengths in the same units as those of the evolutionary distances used to infer the phylogenetic tree. The evolutionary distances were computed using the Poisson correction method and are in the units of the number of amino acid substitutions per site. All positions containing gaps and missing data were eliminated. There were a total of 358 positions in the final dataset. Evolutionary analyses were conducted in MEGA6. Figure S3. Whole genome clustering based on a similarity matrix using the peptidome content, in which amino acid sequences of a genome are converted to tryptic peptides, i.e. the trypti [file 12864_2016_2382_MOESM1_ESM.pdf]

Figure S1. Phylogenetic tree of full-length NarG. Sequences were taken from genomes included in the manuscript as well as all from publically available *B. licheniformis* genomes (dd June 2014) and genomes from other *Bacillus* species, protein ID or locus tag is given between brackets. The evolutionary history was inferred using the Neighbor-Joining method [1]. The optimal tree with the sum of branch length = 1,68972248 is shown. The percentage of replicate trees in which the associated taxa clustered together in the bootstrap test (500 replicates) are shown next to the branches [2]. The evolutionary distances were computed using the Poisson correction method [3] and are in the units of the number of amino acid substitutions per site. All positions containing gaps and missing data were eliminated. There were a total of 1094 positions in the final dataset. Evolutionary analyses were conducted in MEGA6 [4].

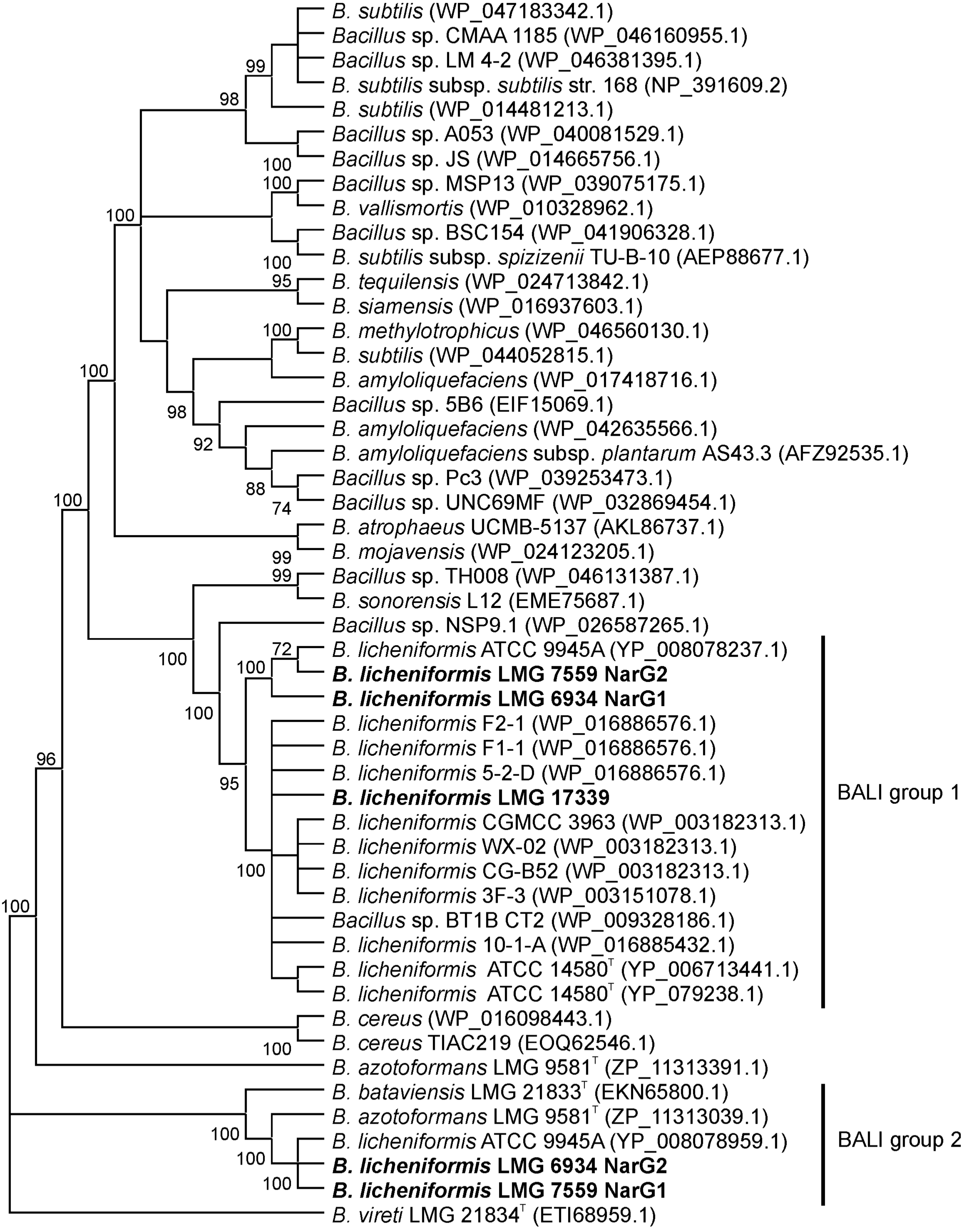
B. subtilis (WP_047183342.1)
Bacillus sp. CMAA 1185 (WP_046160955.1)
Bacillus sp. LM 4-2 (WP_046381395.1)
B. subtilis subsp. subtilis str. 168 (NP_391609.2)
B. subtilis (WP_014481213.1)
Bacillus sp. A053 (WP_040081529.1)
Bacillus sp. JS (WP_014665756.1)
Bacillus sp. MSP13 (WP_039075175.1)
B. vallismortis (WP_010328962.1)
Bacillus sp. BSC154 (WP_041906328.1)
B. subtilis subsp. spizizenii TU-B-10 (AEP88677.1)
B. tequilensis (WP_024713842.1)
B. siamensis (WP_016937603.1)
B. methylotrophicus (WP_046560130.1)
B. subtilis (WP_044052815.1)
B. amyloliquefaciens (WP_017418716.1)
Bacillus sp. 5B6 (EIF15069.1)
B. amyloliquefaciens (WP_042635566.1)
B. amyloliquefaciens subsp. plantarum AS43.3 (AFZ92535.1)
Bacillus sp. Pc3 (WP_039253473.1)
Bacillus sp. UNC69MF (WP_032869454.1)
B. atrophaeus UCMB-5137 (AKL86737.1)
B. mojavensis (WP_024123205.1)
Bacillus sp. TH008 (WP_046131387.1)
B. sonorensis L12 (EME75687.1)
Bacillus sp. NSP9.1 (WP_026587265.1)
B. licheniformis ATCC 9945A (YP_008078237.1)
B. licheniformis LMG 7559 NarG2
B. licheniformis LMG 6934 NarG1
B. licheniformis F2-1 (WP_016886576.1)
B. licheniformis F1-1 (WP_016886576.1)
B. licheniformis 5-2-D (WP_016886576.1)
B. licheniformis LMG 17339
B. licheniformis CGMCC 3963 (WP_003182313.1)
B. licheniformis WX-02 (WP_003182313.1)
B. licheniformis CG-B52 (WP_003182313.1)
B. licheniformis 3F-3 (WP_003151078.1)
Bacillus sp. BT1B CT2 (WP_009328186.1)
B. licheniformis 10-1-A (WP_016885432.1)
B. licheniformis ATCC 14580T (YP_006713441.1)
B. licheniformis ATCC 14580T (YP_079238.1)
BALI group 1
B. cereus (WP_016098443.1)
B. cereus TIAC219 (EOQ62546.1)
B. azotoformans LMG 9581T (ZP_11313391.1)
B. bataviensis LMG 21833T (EKN65800.1)
B. azotoformans LMG 9581T (ZP_11313039.1)
B. licheniformis ATCC 9945A (YP_008078959.1)
B. licheniformis LMG 6934 NarG2
B. licheniformis LMG 7559 NarG1
BALI group 2
B. vireti LMG 21834T (ETI68959.1)
99
98
100
100
100
100
95
100
98
92
88
74
100
99
99
100
72
100
100
95
100
96
100
100
100
100
100

Figure S2. Phylogenetic tree of full-length NarK. Sequences were taken from several *Bacillus* species, as well as reference genomes, locus tag is given between brackets. The evolutionary history was inferred using the Neighbor-Joining method [1]. The optimal tree with the sum of branch length = 6,03046656 is shown. The percentage of replicate trees in which the associated taxa clustered together in the bootstrap test (500 replicates) are shown next to the branches [2]. The tree is drawn to scale, with branch lengths in the same units as those of the evolutionary distances used to infer the phylogenetic tree. The evolutionary distances were computed using the Poisson correction method [3] and are in the units of the number of amino acid substitutions per site. All positions containing gaps and missing data were eliminated. There were a total of 358 positions in the final dataset. Evolutionary analyses were conducted in MEGA6 [4].

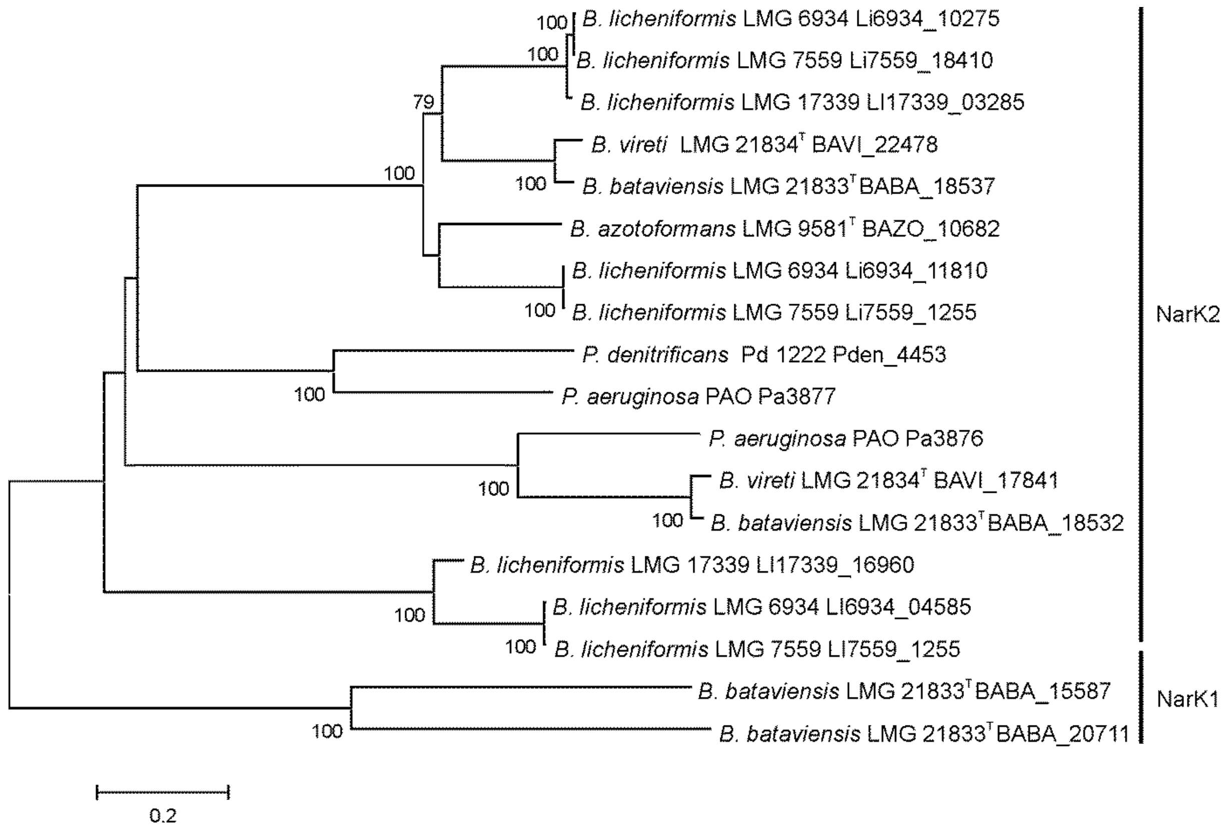

Figure S3. Whole genome clustering based on a similarity matrix using the peptidome content, in which amino acid sequences of a genome are converted to tryptic peptides, i.e. the tryptic peptidome. All publically available *B. licheniformis* genomes (dd June 2014) were included and are designated by their strain number: 9945A (accession number NC_021362) [5], ATCC 14580 (accession number NC_006270) [6], DSM 13 (=ATCC 14580; accession number NC_006322) [7], 5-2-D (accession number NZ_AJLW01000000) [8], F1-1 (accession number NZ_AZSL01000000) [9], 3F-3 (accession number NZ_JFYM01000000) [8], F2-1 (accession number NZ_AZSM01000000) [9], WX-02 (accession number NZ_JH636050) [10], 10-1-A (accession number NZ_AJLV01000001) [11], CGMCC 3963 (accession number NZ_AMWQ01000000) [12], CG-B52 (accession number NZ_AVEZ01000000). Analyses were performed using the Peptidome tool (http://unipept.ugent.be/) [13].

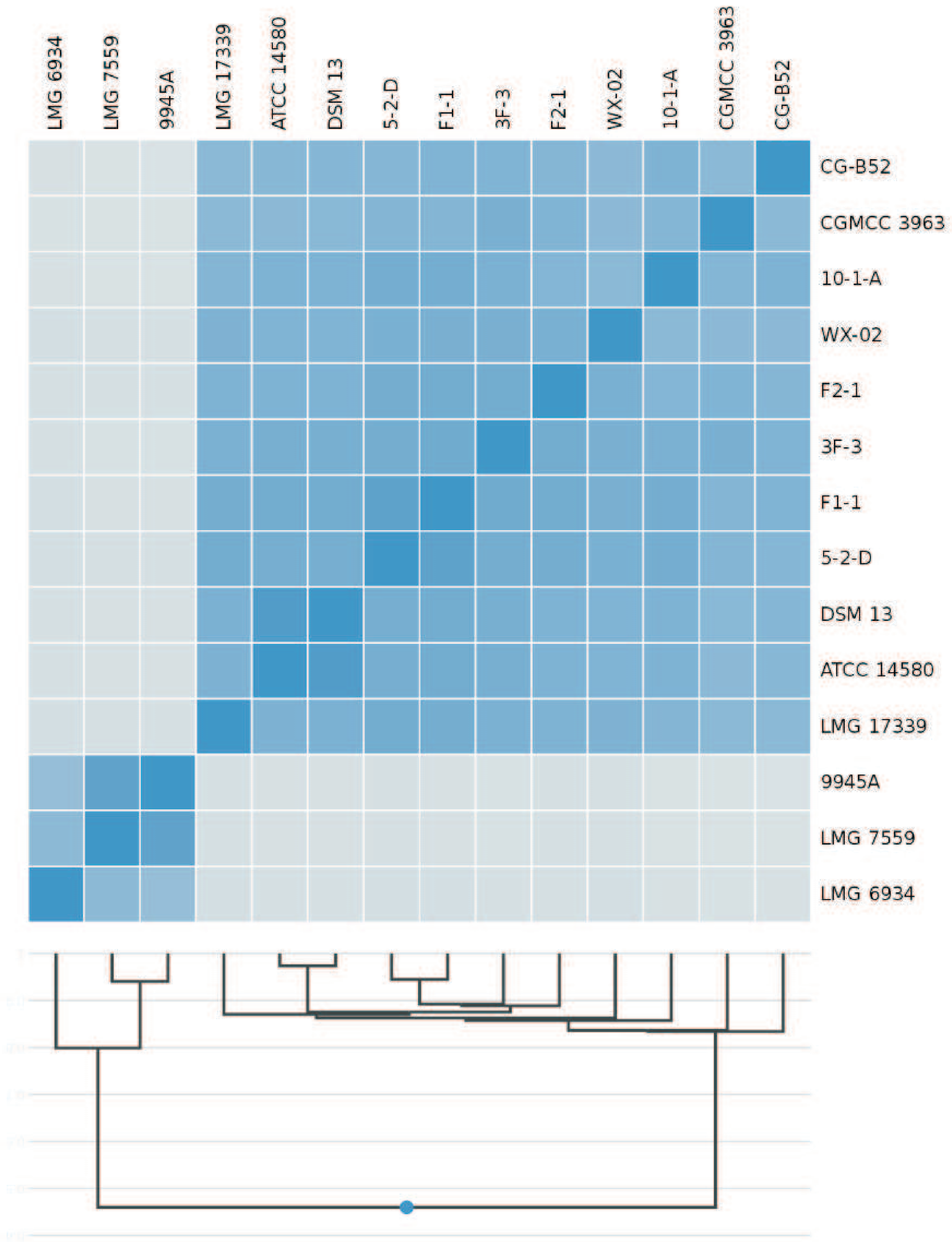
LMG 6934
LMG 7559
9945A
LMG 17339
ATCC 14580
DSM 13
5-2-D
F1-1
3F-3
F2-1
WX-02
10-1-A
CGMCC 3963
CG-B52

REFERENCES

1. Saitou N, Nei M: **The neighbor-joining method: a new method for reconstructing phylogenetic trees**. *Mol Biol Evol* 1987, **4**(4):406-425.
2. Felsenstein J: **Confidence Limits on Phylogenies: An Approach Using the Bootstrap**. *Evolution* 1985, **39**(4):783-791.
3. E. Z, L. P: **Evolutionary divergence and convergence in proteins**. In: *Evolving Genes and Proteins.* Edited by Bryson V, Vogel HJ. New York: Academic Press; 1965: 97-166.
4. Tamura K, Stecher G, Peterson D, Filipski A, Kumar S: **MEGA6: Molecular Evolutionary Genetics Analysis version 6.0**. *Mol Biol Evol* 2013, **30**(12):2725-2729.
5. Rachinger M, Volland S, Meinhardt F, Daniel R, Liesegang H: **First Insights into the Completely Annotated Genome Sequence of *Bacillus licheniformis* Strain 9945A**. *Genome announcements* 2013, **1**(4).
6. Rey MW, Ramaiya P, Nelson BA, Brody-Karpin SD, Zaretsky EJ, Tang M, Leon ALd, Xiang H, Gusti V, Clausen IG *et al*: **Complete genome sequence of the industrial bacterium *Bacillus licheniformis* and comparisons with closely related *Bacillus* species**. *Genome Biology* 2004, **5**:R77.
7. Veith B, Herzberg C, Steckel S, Feesche J, Maurer KH, Ehrenreich P, Bäumer S, Henne A, Liesegang H, Merkl R *et al*: **The Complete Genome Sequence of *Bacillus licheniformis* DSM13, an Organism with Great Industrial Potential**. *J Mol Microbiol Biotechnol* 2004, **7**:204-211.
8. Li L, Wang Y, Wang K, Li K, Ma C, Xu P: **Genome Sequence of Thermophilic Bacillus licheniformis Strain 3F-3, an Efficient Pentose-Utilizing Producer of 2,3-Butanediol**. *Genome announcements* 2014, **2**(3).
9. Dhakal R, Seale RB, Deeth HC, Craven H, Turner MS: **Draft Genome Comparison of Representatives of the Three Dominant Genotype Groups of Dairy *Bacillus licheniformis* Strains**. *Appl Environ Microbiol* 2014, **80**(11):3453-3462.
10. Yangtse W, Zhou Y, Lei Y, Qiu Y, Wei X, Ji Z, Qi G, Yong Y, Chen L, Chen S: **Genome sequence of Bacillus licheniformis WX-02**. *J Bacteriol* 2012, **194**(13):3561-3562.
11. Li L, Su F, Wang Y, Zhang L, Liu C, Li J, Ma C, Xu P: **Genome sequences of two thermophilic Bacillus licheniformis strains, efficient producers of platform chemical 2,3-butanediol**. *J Bacteriol* 2012, **194**(15):4133-4134.
12. Wu Q, Peng S, Yu Y, Li Y, Xu Y: **Genome Sequence of Bacillus licheniformis CGMCC3963, a Stress-Resistant Strain Isolated in a Chinese Traditional Solid-State Liquor-Making Process**. *Genome announcements* 2013, **1**(1).
13. Mesuere B, Devreese B, Debyser G, Aerts M, Vandamme P, Dawyndt P: **Unipept: tryptic peptide-based biodiversity analysis of metaproteome samples**. *Journal of proteome research* 2012, **11**(12):5773-5780.
